# Occult Fat in Faeces

**Published:** 1915-01

**Authors:** 


**Occult Fat in Faeces.** Saathoff of Obersdorf, The Hospital of London, slightly condenses a smear by holding the slide over a Bunsen or alcohol flame. He adds 90% of glacial acetic acid in absolute alcohol containing Sudan II and mixes with the end of a match. Examined warm, the fat is visible as yellow or red drops. On cooling, acicular crystals of fatty acids are seen which disappear on again warming. Normal dried faeces contain about 20% of fat or 6 grams per day. Increase is noted in thyroid disease, hepatic lesions, pancreatic failure and certain intestinal disturbances.

